# Inhibition of enteric methanogenesis in dairy cows induces changes in plasma metabolome highlighting metabolic shifts and potential markers of emission

**DOI:** 10.1038/s41598-020-72145-w

**Published:** 2020-09-24

**Authors:** Bénédict Yanibada, Ulli Hohenester, Mélanie Pétéra, Cécile Canlet, Stéphanie Durand, Fabien Jourdan, Julien Boccard, Cécile Martin, Maguy Eugène, Diego P. Morgavi, Hamid Boudra

**Affiliations:** 1 Université Clermont Auvergne, INRAE, VetAgro Sup, UMR Herbivores, Saint-Genès-Champanelle, Clermont-Ferrand, France; 2 Université Clermont Auvergne, INRAE, UNH, Plateforme d’Exploration du Métabolisme, MetaboHUB Clermont, 63000 Clermont-Ferrand, France; 3 Toxalim, Research Centre in Food Toxicology, Université de Toulouse, INRAE, ENVT, INP-Purpan, UPS, 31027 Toulouse, France; 4 Axiom Platform, MetaToul-MetaboHUB, National Infrastructure for Metabolomics and Fluxomics, 31027 Toulouse, France; 5 Institute of Pharmaceutical Sciences of Western Switzerland, University of Geneva, Geneva, Switzerland

**Keywords:** Biological techniques, Chemical biology, Biomarkers

## Abstract

There is scarce information on whether inhibition of rumen methanogenesis induces metabolic changes on the host ruminant. Understanding these possible changes is important for the acceptance of methane-reducing practices by producers. In this study we explored the changes in plasma profiles associated with the reduction of methane emissions. Plasma samples were collected from lactating primiparous Holstein cows fed the same diet with (Treated, n = 12) or without (Control, n = 13) an anti-methanogenic feed additive for six weeks. Daily methane emissions (CH_4_, g/d) were reduced by 23% in the Treated group with no changes in milk production, feed intake, body weight, and biochemical indicators of health status. Plasma metabolome analyses were performed using untargeted [nuclear magnetic resonance (NMR) and liquid chromatography-mass spectrometry (LC–MS)] and targeted (LC–MS/MS) approaches. We identified 48 discriminant metabolites. Some metabolites mainly of microbial origin such as dimethylsulfone, formic acid and metabolites containing methylated groups like stachydrine, can be related to rumen methanogenesis and can potentially be used as markers. The other discriminant metabolites are produced by the host or have a mixed microbial-host origin. These metabolites, which increased in treated cows, belong to general pathways of amino acids and energy metabolism suggesting a systemic non-negative effect on the animal.

## Introduction

Methane emitted by domestic ruminants is a major worldwide contributor to greenhouse gases released annually from anthropogenic sources^1^. The production of methane in the gastrointestinal tract of ruminants is extensively studied and different strategies are being tested to mitigate methane emissions from this source. The main strategies under development are based on changes in diet composition^2^, the use of feed additives, e.g.^3,4^, the modulation of the rumen microbiota, e.g.^5^ and genetic selection for low methane-emitting animals^6^. Great advances are being made for these different strategies and some of them can already be applied in the field. However, some critical points still need to be addressed before these solutions can be widely recommended to and adopted by producers.

The reduction of methane production in the gastrointestinal tract of ruminants theoretically increases the amount of energy from feeds that is available for productive purposes. However, one aspect that hamper the adoption of methane-mitigation strategies, particularly additives, is that inhibiting rumen methanogenesis usually does not translate into improved production^7^. Some of the energy spared when methane production is inhibited can be explained by the increase in the amount of dihydrogen expelled and changes in fermentation end products but most energy remains unaccounted^7,8^. Although a part of this unaccounted energy should benefit the host animal, observations may be elusive if circumscribed to an immediate increase in milk production or weight gain, as stated above. To this end, the use of metabolomics can bring a more refined picture of any potential metabolic impact.

Other important issue is that methane emissions have to be measured to evaluate the effectiveness of different strategies. Under controlled conditions available in experimental research stations the use of respiration chambers, gas tracer techniques and repeated spot measurement techniques are commonly implemented^9,10^. These methods are accurate and repeatable but although some of them can be applied in farms, their use is not adapted to a high number of animals. Other limitations are the cost, the invasive character for some of them and the required technicity that makes their utilization difficult to implement in farms. To address these issues, alternative methods to estimate methane emission using indirect markers or proxies are tested^11^. Among them, the analysis of milk fatty acids and mid-infrared (MIR) profiles seems to be promising alternatives^12–15^. However, this is limited to lactating dairy cows whereas a large proportion of enteric methane emissions from ruminants is originated from beef production and growing replacement dairy heifers.

Metabolomics is an approach that has shown utility for the discovery of metabolic functions and metabolites associated with various conditions such as disease, stress and nutrition. Blood, particularly plasma, is a biological fluid that has been widely studied in metabolomics^16–19^. It has the advantage of being routinely obtainable from all types of ruminants. In cattle, a plasma metabolomic approach was successfully used to assess performance efficiency in fattening steers^20^, heat stress^21^ and metabolic disorders such as clinical and subclinical ketosis^22^ in lactating dairy cows. Plasma metabolites reflect both the activity of gut microbes and the host animal metabolism^20,23^. The characterization of potential plasma changes induced by reduced enteric methane emissions can bring new insights on the integrated microbial-host metabolism. This type of information can be translated into practical applications that use plasma metabolites as proxies for evaluating enteric methane emissions and assessing metabolic fitness in ruminant production system.

The objective of this study was to explore changes in plasma profiles associated with the reduction of methane emissions. We used primiparous dairy cows in the same physiological state that were fed (n = 12) or not (n = 13) a methane inhibitor that specifically blocks the last enzymatic step of the methanogenesis pathway unique to methanogenic archaea. This last step, catalysed by the enzyme methyl-coenzyme M reductase, is common to all rumen methanogens independently of the substrate utilization (hydrogenotrophic, methylotrophic or acetogenic)^24,25^. These kind of inhibitors do not have a direct effect on other microbial groups or on host cells^26–28^. We hypothesized that plasma metabolites that originate from rumen microbes, from the host or both (co-metabolites) will be impacted by a reduction of methane emissions in the rumen.

## Results

### Enteric methane reduction without major changes in performances and blood parameters

The anti-methanogenic additive reduced enteric methane emissions (CH_4_ g/day) by 23% without affecting dry matter intake and milk production (Table 1). Treated cows, however, tended to have higher concentration of milk protein (P = 0.078) and lower concentration of lactose (P = 0.053). Feed intake increased from week 1 up to week 5 but this increase was similar in both groups and it was related to the lactation stage. Biochemical analyses performed on plasma samples for monitoring general health aspects showed values within normal ranges for dairy cows^29^. This indicates no overt health problems that could have influenced the metabolome and confirms the absence of negative effects of the treatment. Non-esterified fatty acids (NEFA) tended to decrease in treated cows (P = 0.099) (Table 1). Some metabolites and enzymatic activities differed between the beginning and end of the experimental period but, as for intake, these changes were not related to treatment.

**Table 1:** Nutrient intake, milk yield and composition, enteric methane production and plasma biochemical analysis in lactating dairy cows fed a diet supplemented (Treated, n = 12) or not (Control, n = 13) with an anti-methanogenic compound.

Item	Control		Treated		SEM	P-value		
Week of study	1	5	1	5		Treatment	Week	Treat × wk
**Nutrient intake**								
DMI (kg/day)	18.0	19.5	18.5	18.8	0.44	0.758	0.031	0.129
**Milk yield and composition**								
Milk yield (kg/day)	24.6	23.9	24.8	24.4	0.93	0.772	0.060	0.617
ECM (kg/day)^a^	22.3	21.5	22.8	22.7	0.81	0.461	0.231	0.256
Fat (g/kg)	35.2	33.8	35.4	35.4	1.24	0.579	0.337	0.328
Protein (g/kg)	28.1	29.9	29.4	31.2	0.55	0.078	< 0.0001	0.956
Lactose (g/kg)	51.8	51.7	50.6	51.2	0.38	0.053	0.445	0.249
Somatic cell count (× 10^3^/ml)	147.4	145.4	152.9	89.5	37.74	0.581	0.248	0.276
**Methane**^b^								
CH_4_ production (g/day)	–	334.9	–	259.0	16.5	0.006		
CH_4_ yield (g/kg DMI)	–	17.4	–	13.6	0.65	0.001		
CH_4_ intensity (g/kg ECM)		14.0		10.5	0.66	0.002		
**Plasma biochemical parameters**^a^								
Urea (mmol/L)	0.16	0.20	0.17	0.20	0.010	0.759	< 0.0001	0.231
Glucose (mmol/L)	0.51	0.51	0.48	0.52	0.017	0.597	0.272	0.254
BetaOH (µmol/L)	0.40	0.30	0.38	0.29	0.026	0.666	0.002	0.712
NEFA (mmol/L)	0.29	0.23	0.23	0.18	0.033	0.099	0.085	0.997
GGT (IU)	22.3	22.3	21.2	23.6	1.18	0.950	0.049	0.055
PAL (IU)	107	120.6	110.2	121.6	10.07	0.884	0.032	0.884
ALAT (IU)	22.6	25.3	23.4	23.7	1.47	0.840	0.115	0.207
ASAT (IU)	70.2	71.6	69.5	71.4	5.68	0.949	0.627	0.949

^a^ECM, energy corrected milk (0.2595 * milk yield (kg/day) + 12.55 * fat (g/kg) + 7.39 * protein (g/kg)).

^b^Enteric methane emission was measured on 16 cows (8 per group) in open circuit respiration chambers in week 5 of the treatment period.

^c^Normal range of values of biochemical parameters from (Bellier, 2010 and Synlab, 2010): urea 3–8 mmol/L; glucose 2.5–4.2 mmol/L; beta hydroxybutyrate (BetaOH) < 1,400 µmol/L; non esterified fatty acid (NEFA) 0–0.45 mmol/L; gamma glutalyl transpeptidase (GGT) 6–18 IU; phosphatase alkaline (PAL) < 500 IU ; alanine amino transferase (ALAT) 11–40 IU; asparate amino transferase (ASAT) 78–132 IU.

*SEM* standard error of the mean.

### Plasma metabolome differs between high and low methane-emitting cows

The two groups of cows used in this study received the same diet and were comparable in terms of age, body weight, days in milk and milk production. All these aspects are major metabolomic determinants that were controlled in the study. In contrast, as low methane emission was the only macroscopic phenotypic trait induced by the treatment, we only expected subtle changes in the metabolome. Consequently, variable selection approaches were used in order to highlight relevant metabolites of interest and reduce the complexity of statistical models^30^. To have a greater coverage of the metabolome, we used untargeted and targeted approaches. For the untargeted, NMR and LC–MS were used, whereas LC–MS/MS was used for the targeted method.

#### Untargeted plasma metabolome analyses

**Figure 1:**
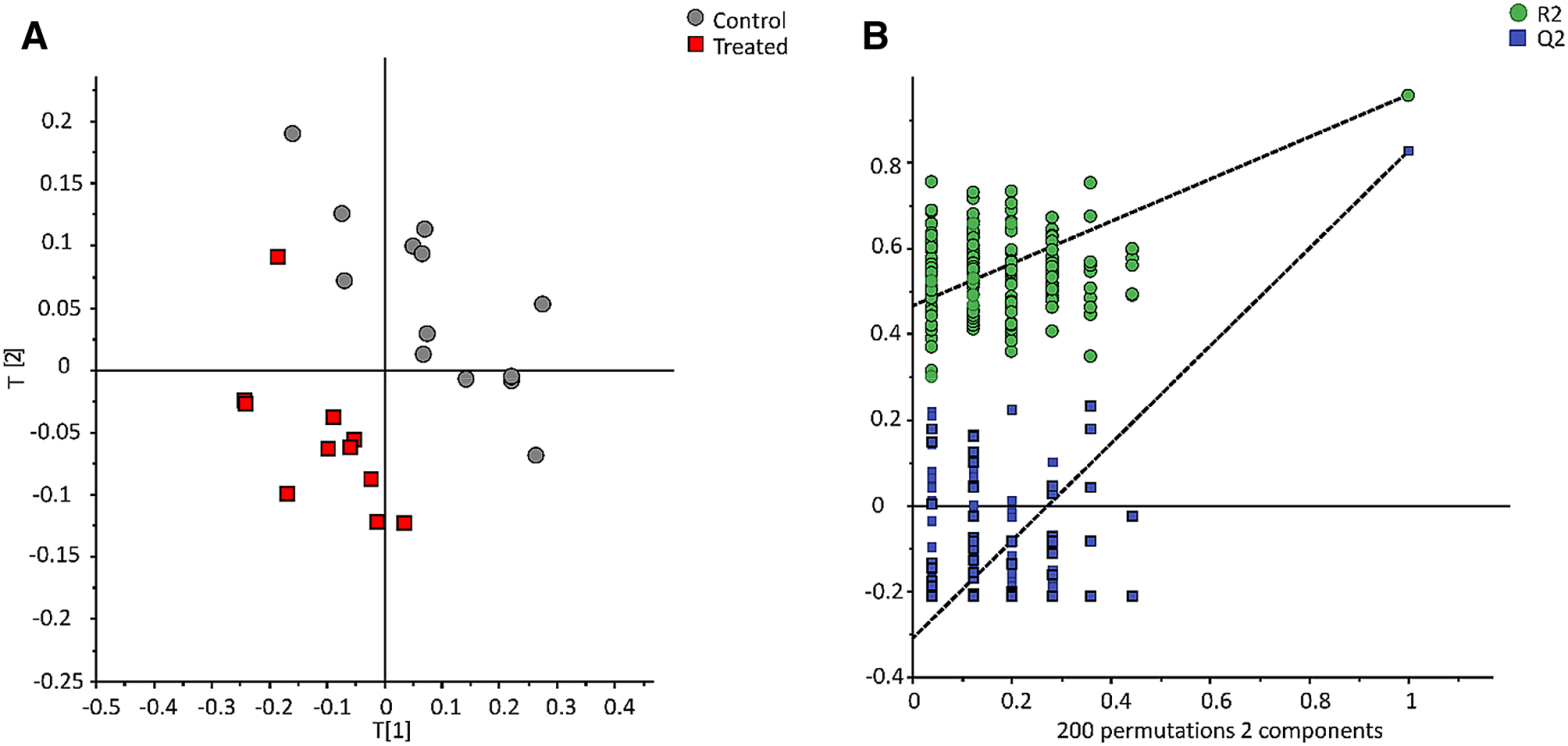
Orthogonal signal correction partial least square discriminant analysis (OSC-PLS-DA) of NMR data (891 variables; n = 2; R2X = 0.41; R2Y = 0.854; Q2 = 0.55) (left panel) and permutation test of the model (right panel). Control group (gray circle, n = 13) vs Treated group (red square, n = 12).

For the NMR dataset (n = 891 variables after processing), the orthogonal signal correction (OSC) ^31^ filtering was applied for removing features that were not related to group discrimination. The OSC PLS-DA model with one component removed, explained 55.3% of the variability (n = 2; R^2^X = 0.41; R^2^Y = 0.854; Q^2^ = 0.55) (Fig. 1). Out of 120 variables with importance in the projection (VIP) value higher than 1.2, 17 metabolites were annotated by querying an in-house database. Among them, 13 were identified (level 1) by comparing one dimensional and two-dimensional spectra with standard compounds spectra analysed on the same spectrometer (Table 2).

**Table 2:** Discriminant plasma metabolites identified by NMR in lactating dairy cows fed a diet supplemented (n = 12) or not (n = 13) with an anti-methanogenic compound.

Metabolites	Chemical shift (ppm)	Multiplicity^a^	P value	VIP value^b^	Annotation level	Fold change^c^
**Amino acids**						
Asparagine*	2.925	dd	0.05	1.1	1	1.05
Betaine	3.275	s	0.05	2.3	1	0.95
Creatine	3.035	s	0.11	4.0	2	1.13
Creatinine	4.055	s	0.07	1.5	2	1.12
Glutamine*	2.445	m	0.16	2.2	1	1.15
Glycine*	3.565	s	0.02	5.7	1	1.22
Isoleucine	1.005	d	0.12	2.7	1	0.83
Leucine	1.685	m	0.16	1.3	1	0.91
Methionine*	2.135	m	0.02	2.5	2	1.11
Threonine*	4.235	m	0.16	1.4	1	1.05
Valine*	1.055	d	0.13	2.7	2	0.60
**Carbohydrates**						
Glucose	3.265	ddt	0.43	3.5	1	1.14
**Carboxylic acids**						
Acetic acid	1.925	s	0.22	3.9	1	0.61
Citric acid	2.515	d		2.0	1	1.19
Formic acid	8.455	s	0.05	1.7	1	2.18
Lactic acid	1.345	d	0.27	2.5	1	0.79
**Other**						
Dimethyl sulfone	3.155	s	0.005	6.1	1	1.92

^a^Multiplicity: s = singlet; d = doublet; dd = doublet of doublet; m = multiplet; t = triplet.

^b^Variable importance in the projection. For multiple chemical shifts, the highest VIP value is given.

^c^Fold change was calculated as the average value of the treated group to that of the control.

*Metabolite also identified with another analytical platform (targeted or untargeted LC-QToF-MS) used in this study.

**Figure 2:**
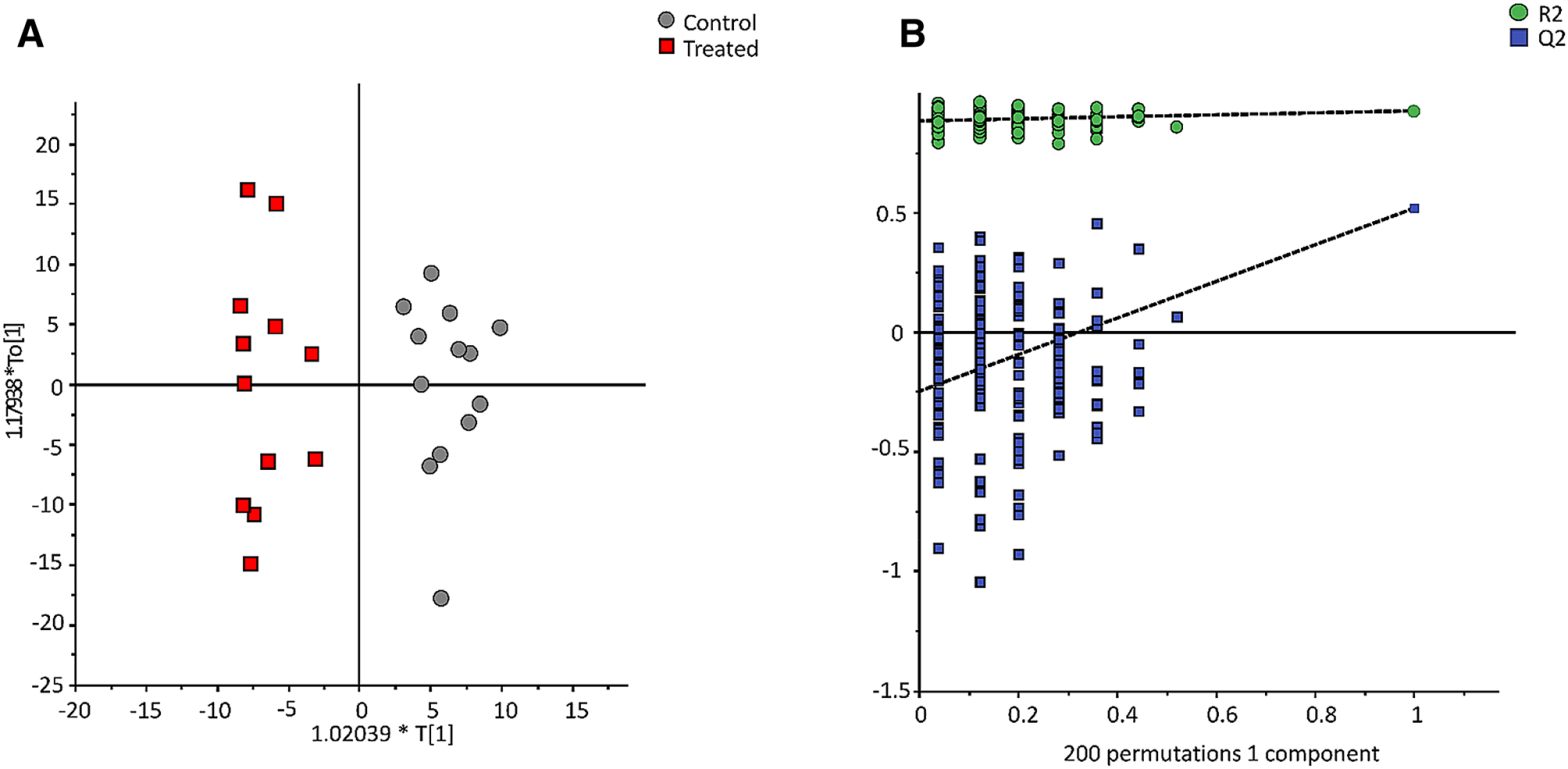
Orthogonal projection to latent structures-discriminant analysis (OPLS-DA) model of MS data after a variable selection based on VIP values > 0.5 (559 of the 771 variables; n = 1 + 1 + 0; R2X = 0.21 R2Y = 0.93; Q2 = 0.52) (left panel) and permutation test of the model (right panel). Control group (gray circle, n = 13) vs Treated group (red square, n = 12).

For MS data, 771 variables were obtained and an OPLS-DA model was built after removing 212 variables with a VIP value less than 0.5. The obtained model has an acceptable predictive capacity (Q^2^ = 0.52) (Fig. 2) and 108 variables with VIP value higher than 1.2 were selected as discriminant variables. Table 3 shows the eight metabolites that were identified. Methylhistidine, methionine, pyrrolidine, and stachydrine, all related to amino acids and derivatives, were confirmed by analytical standards (level 1). The remaining metabolites were identified on the basis of their fragmentation and adduct patterns (level 2) as trihydroxy-cholanoic acid derivatives (Supplementary Table . S1).

**Table 3:** Discriminant plasma metabolites identified by untargeted LC–MS in lactating dairy cows fed a diet supplemented (n = 12) or not (n = 13) with an anti-methanogenic compound.

Metabolites	Formula	m/z	Δm/z (ppm)	Rt	P value	VIP value	Annotation level	Fold change^a^
Methylhistidine	C_7_H_12_ N_3_O_2_	170.0925	− 0.14	0.8	0.500	1.2	1	1.0
Stachydrine	C_7_H_13_NO_2_	144.1019	0	1.0	0.040	1.4	1	1.4
Pyrrolidine	C_4_H_10_N	72.0806	0	1.2	0.110	1.4	1	0.9
Methionine*	C_5_H_12_NO_2_S	150.0583	− 0.24		0.003	1.6	1	1.2
L-alpha-amino-1H-pyrrole-1-hexanoic acid	C_10_H_17_N_2_O_2_	197.1285	0	–	0.002	1.5	2	1.3
Trihydroxy-cholanoic acids and derivates	–	–	0	–	0.006	1.8	2	1.2
LysoPC (18:2)	C_26_H_51_NO_7_P	523.3486	0	14.6	0.290	1.3	2	0.9
PC(14:0)	C_22_H_47_NO_7_P	468.3086	2.80	13.8	0.190	1.3	2	0.9

*Rt*  retention time, *VIP* variable importance in the projection.

^a^Fold change was calculated as the average value of the treated group to that of the control.

*Metabolite identified with another analytical platform (untargeted NMR).

#### Targeted plasma metabolomics analysis

**Figure 3:**
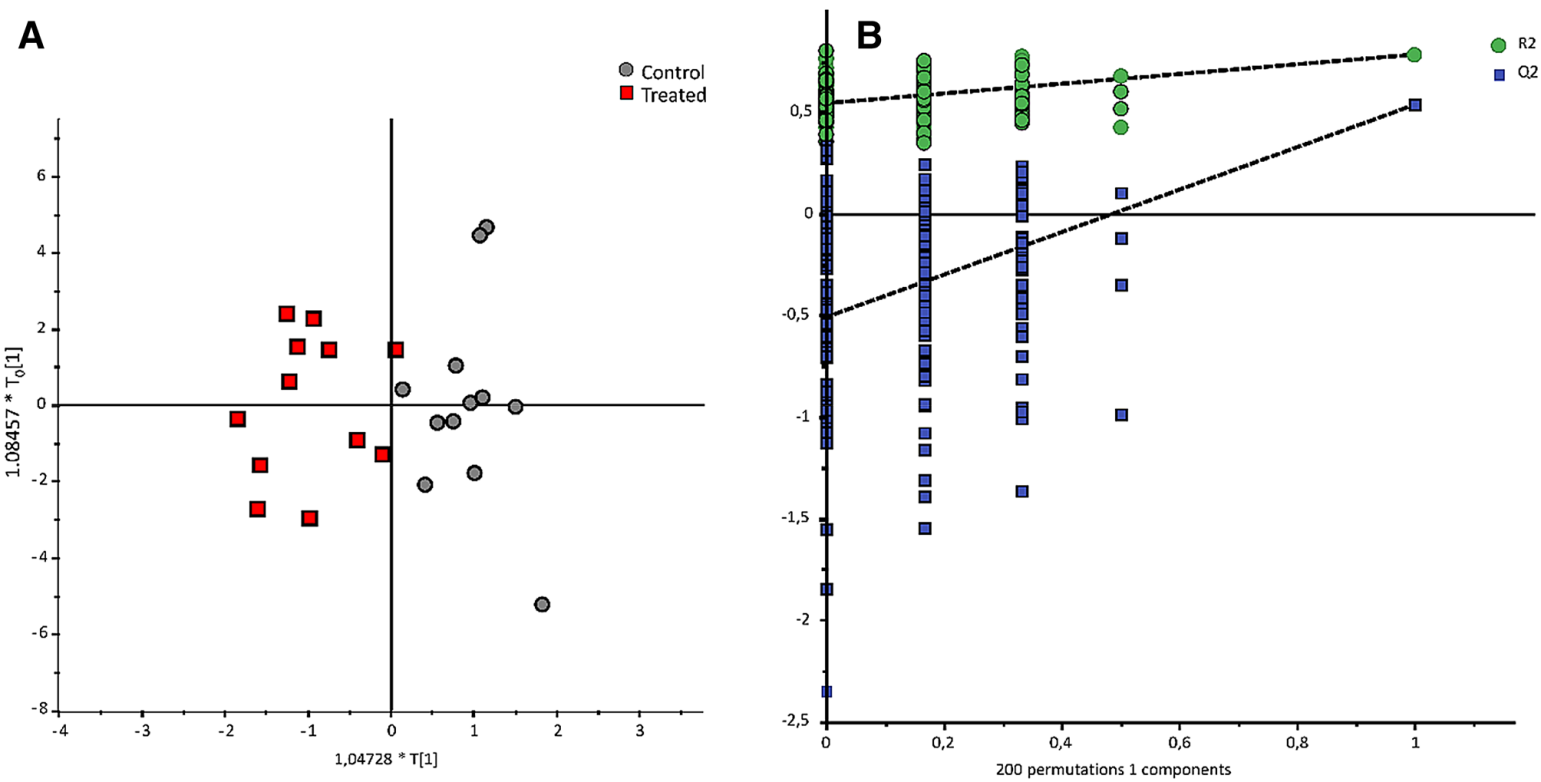
Orthogonal projection to latent structures-discriminant analysis (OPLS-DA) of the LC–MS targeted metabolomics analysis. (n = 2; R2X = 0.46 R2Y = 0.78; Q2 = 0.54) (left panel) and permutation test of the model (right panel). Control group (gray circle, n = 12 vs Treated group (red square, n = 12). A sample was discarded from the control group due to a technical issue.

The LC–MS/MS method used targets 188 metabolites (*Absolute IDQ p180 Kit,* Biocrates Life Sciences AG, Austria). The statistical analysis was done on 107 metabolites that had levels above the limit of quantification (see Supplementary Table S2). The obtained OPLS-DA model has an acceptable predictive ability (Q^2^ = 0.54) (Fig. 3), and 29 discriminant metabolites with VIP values higher than 1.2 were selected (Table 4). These 29 metabolites were for the most part amino acids and derivatives that increased in the Treated group and glycerophospholipids that decreased.

**Table 4:** Discriminant plasma metabolites identified by targeted LC–MS in cows fed a similar diet supplemented (Treated, n = 12) or not (Control, n = 13) with an anti-methanogenic compound.

Metabolite	P value	VIP value	Fold change^a^
**Amino acids and derivatives**			
Arginine	0.08	1.55	1.15
Asparagine*	0.05	1.61	1.17
Citrulline	0.08	1.39	1.16
Glutamine*	0.11	1.32	1.15
Glycine*	< 0.0001	2.68	1.32
Methionine*	0.11	1.29	1.12
Proline	0.10	1.52	1.21
Serine	< 0.0001	2.69	1.36
Threonine*	0.23	1.16	1.12
Tyrosine	0.19	1.63	1.15
Valine*	0.07	1.28	0.87
Kynurenine	0.16	1.44	1.17
Symmetric dimethylarginine	0.08	1.37	1.14
Serotonin	0.008	3.24	2.24
Taurine	0.10	1.41	1.28
**Acylcarnitines**			
Propionylcarnitine	0.50	1.18	1.07
Valerylcarnitine	0.28	1.59	1.11
**Glycerophospholipids**			
PC aa C34:3	0.06	1.98	0.86
PC aa C34:4	0.10	1.49	0.82
PC ae C30:1	0.03	1.87	0.82
PC ae C32:2	0.04	1.55	0.85
PC ae C34:2	0.07	1.32	0.87
PC ae C34:3	0.02	1.98	0.79
PC ae C36:4	0.02	1.82	0.80
PC ae C36:5	0.01	1.96	0.79
PC ae C38:5	0.08	1.21	0.86
PC ae C38:6	0.02	1.74	0.81
**Sphingolipids**			
SM C20:2	0.04	4.51	2.39
SM C26:1	0.18	1.18	1.18

*VIP*  variable importance in the projection.

^a^Fold change was calculated as the average value of the treated group to that of the control.

*Metabolites also identified with another analytical platform (NMR or untargeted LC-QToF-MS) used in this study.

#### Associations between discriminant metabolites

**Figure 4:**
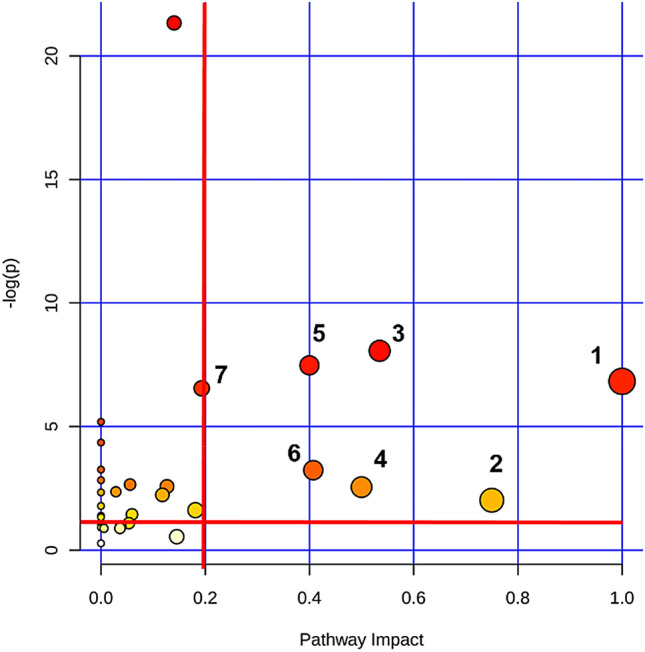
Plot showing modified pathways in the Treated group. From the 48 identified discriminant metabolites, 41 metabolites were mapped on *Bos taurus* network for pathway enrichment. These included generic identifiers for sphingomyelin (n = 2) and phosphatidylcholine (n = 11). The modified pathways are: **1/**Valine, leucine and isoleucine biosynthesis **2/**Taurine and hypotaurine metabolism **3/**Glycine, serine and threonine metabolism **4/**Phenylalanine, tyrosine and tryptophan biosynthesis **5/**Methane metabolism **6/**Glyoxylate and dicarboxylate metabolism **7/**Arginine and proline metabolism. The plot was built based on the pathway enrichment analysis (node colors) and on the pathway impact values resulting from the pathway topology analysis (node size). The red lines correspond to a threshold of – log p = 1.5 and a pathway impact = 0.2.

**Figure 5:**
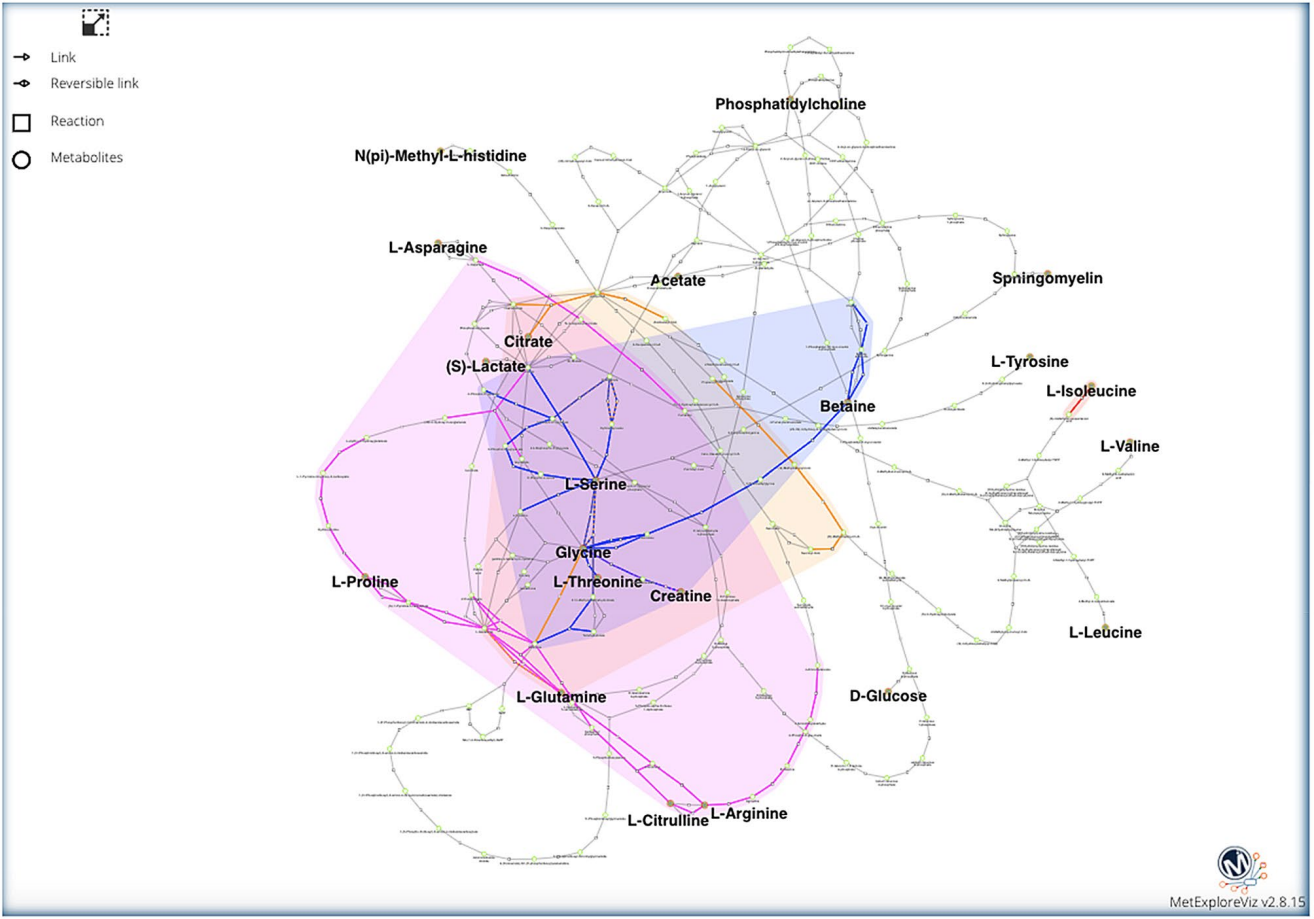
Extracted subnetwork showing the link between 21 metabolites (bold lettering) mapped in Metexplore version 2.20 (https://metexplore.toulouse.inra.fr/metexplore2.20.12/) which implements MetExploreViz package^79^. The network used in MetExplore is KEGG version of Bos Taurus (MetExplore biosource id 2952, corresponding to KEGG database version of May 4 2015). Pathways enriched with Metaboanalyst are coloured: arginine and proline metabolism (purple); glyoxylate and dicarboxylate metabolism (yellow); glycine, serine and threonine metabolism (blue); valine, leucine and isoleucine metabolism (red).

To identify metabolic pathways that were impacted in low-emitters cows, discriminant metabolites identified by the three analytical platforms (n = 48) were mapped onto the *Bos taurus* network using KEGG identifiers as implemented in MetaboAnalyst^32^. Figure 4 shows that seven metabolic pathways differed between control and treated groups. In addition, we performed a subnetwork analysis that links processes spanning several metabolic pathways (MetExplore^33^). Using the *Bos taurus* network and KEGG identifiers, we highlighted 21 interconnected metabolites in the extracted subnetwork (Fig. 5; Supplementary Table S3 to S5).

## Discussion

The reduction of enteric methane emissions from ruminants is of concern for the sustainability of ruminant production systems. For monitoring the effectiveness of different reduction strategies and for testing and validating new experimental treatments, it is both essential to assess methane emissions and to understand any indirect effect of methane reduction on the host animal metabolism. The aim of this study was twofold: (i) to explore metabolic changes associated with a reduction in methane emissions in cattle and (ii) to identify plasma metabolites that could be used as biomarkers of enteric methane production. To maximize the chance of identifying potential biomarkers of methane emissions in plasma, we used a specific anti-methanogenic compound that inhibits the last step of methanogenesis and analyzed plasma samples using three analytical platforms. The combination of untargeted (NMR and MS) and targeted MS methods allowed to enlarge the coverage of the metabolome. Some discriminant metabolites (asparagine, glutamine, glycine, methionine, threonine and valine) were identified with at least two analytical platforms, which reinforces their importance.

This study was performed under controlled conditions, involving healthy dairy cows in the same lactation stage and fed the same diet. We think that these conditions were required to observe specific changes in the metabolome of low methane-emitting animals and identify potential marker metabolites. Between 20 to 40% reduction in methane emissions can be observed in animals receiving dietary treatments or that were selected for low emission^34,35^. This amount of methane reduction is not expected to produce massive changes in the metabolome as those reported for diseases^36^. Notwithstanding, and although fold change differences in discriminant metabolites were moderate, a good separation was observed between treated and control cows.

The majority of the annotated discriminant metabolites are amino acids that predominantly increased in the Treated group. Higher plasma concentration of amino acids is associated to greater availability for milk protein synthesis^37^ that agrees with the tendency for higher milk protein observed in this study. In addition, higher plasma concentration of amino acids may indicate a greater synthesis of microbial protein in the rumen^38^ and methane inhibition has been shown to improve the efficiency of microbial protein in vitro^39^. The concentration of glycine, serine and threonine increased in Treated cows. These amino acids and related metabolites creatine and creatinine, belong to the glycine, serine and threonine metabolism pathway^40^. Serine, a non-essential amino acid is an intermediate of the glycolysis pathway, whereas glycine, an amino acid derived from serine is involved in the production of DNA and phospholipids and in cell energy metabolism. Creatine is mainly synthetized in the liver from different amino acids like glycine, arginine and methionine. Threonine an essential amino acid supplied by rumen microbes or the diet can be catabolized to glycine by mean of the action of threonine dehydrogenase. Creatine and its natural breakdown product, creatinine, are involved in cell energy metabolism^22^. Levels of branched essential amino acids isoleucine and valine decreased in the treated group whereas leucine increased. These amino acids, supplied by rumen microbes or by the diet, have an important role in the regulation of amino acids and protein metabolism in mammals^41,42^. Subnetwork analysis shows that isoleucine, leucine and valine are closely related to the same pathway. Explaining the differences between groups for these branched amino acids or whether the differences between them are due to increased biosynthesis or degradation will require further targeted analysis. This can be achieved for instance by exploiting the list of reactions belonging to the sub-network connecting the discriminant metabolites (see supplementary table S5). Nevertheless, it has been reported that increased levels of rumen propionic acid (see below) and glucose, as has been observed in this work, are associated with lower concentrations of branched-chain amino acids in cows plasma^43,44^.

Based on the overall trends discussed above, we can hypothesize that changes in amino acids concentration in the Control and Treated groups are partly due to changes in rumen microbial activity. Sustaining this suggestion are the changes observed in acetic acid and glucose. Acetic acid, one of the main volatile fatty acids synthesized by ruminal microbes during the fermentation of carbohydrates^45^, was lower in the Treated group compared to controls. Ruminal production of acetic acid releases dihydrogen that is associated with increased methanogenesis. In contrast, propionic acid production consumes dihydrogen and is associated to decreased methanogenesis^46,47^. Propionic acid absorbed from the rumen is the major substrate for hepatic neoglucogenesis in ruminants^48^ and it is positively associated with glucose in plasma^49^. In our study, cows in the Treated group had greater concentration of glucose, although the fold change was minor. Correspondingly, the rumen of these Treated cows had greater proportion of propionic acid and lesser proportion of acetic acid that the Control group (unpublished data).

The changes observed in phosphatidylcholines, acylcarnitines and sphingomyelins in the Treated group are similar to those described for cows with low lipolysis in comparison with cows with high lipid mobilization^50^. A greater concentration of acylcarnitines in plasma have been related to less inflammation, better liver function and improved health in dairy cows^51^. Branched-chain amino acids are the source of odd-chain propionylcarnitine and valerylcarnitine^52^ highlighted in this work. These plasma metabolites are in accord with the lower concentration of NEFA observed for the Treated group. Altogether, these changes suggest an improved nutritional balance when methane emissions were reduced together with a greater availability of energetic compounds and amino acids.

Some metabolites identified in this study might have a more direct association with methane emissions. To begin with, the pathway of methane metabolism was enriched in MetaboAnalyst. However, we could not get additional insight using MetExplore as was the case for other pathways. MetExplore uses curated metabolic networks from a single organism and the methanogenesis pathway that is performed by methanogens is logically not available in *Bos taurus*. The concentration of methionine, an essential amino acid supplied by the diet or synthesized in the rumen^53^ increased in the Treated group. Methionine and other metabolites related to this amino acid identified in this study are hypothetically related to methanogenesis. In the rumen, methionine is slowly degraded compared to other amino acids and the main initial product of degradation is methanethiol^54^. Methanethiol is at the origin of dimethylsulfone and formic acid, two metabolites with higher abundance in treated cows. These metabolites can be either produced by microbial metabolism or microbial-mammalian co-metabolism^55^. Conversion of methanethiol to formic acid produces hydrogen sulfide and consumes hydrogen. In the rumen, this conversion is performed by anaerobic sulphate-reducing microbes that efficiently compete with methanogens for hydrogen utilization^25^. Concurring with our results, inhibition of enteric methanogenesis by chloroform or 3-NOP additives increased the amount of dimethylsulfone in the rumen^56^. Similarly, in lake ecosystems, inhibition of methanogenesis by chloroform resulted in accumulation of methanethiol^57^. These inhibitors specifically blocks the last enzymatic step of the methanogenesis pathway. In addition, about 18% of the rumen methane is produced from formic acid that acts as an electron donor^25,58^. Formic acid is readily utilised in the rumen by methanogens but its concentration increased when methane production was inhibited^8,59^. This is accord with the greater plasma concentration of formic acid observed in this work in treated cows suggesting a lesser uptake by methanogens in the rumen.

Tryptophan is an essential amino acid that is metabolized along two pathways producing two metabolites that also increased in treated cows: serotonin and kynurenine. The latter, which is involved in the production of nicotinamide adenosine dinucleotide (NAD) accounts for 95% of total body tryptophan metabolism. Kynurenine is the first stable intermediate that is formed in the kynurenine pathway^60^. The other metabolite is the neurotransmitter serotonin that is mainly found in the gastrointestinal tract and is associated to gut motility in mammals. Interestingly, serotonin has been negatively related to methane emission in humans^61,62^. The rumen is rich in serotonin receptors where it increases the tonus and volume of gas eructated while decreasing secondary contractions^63,64^. In contrast, it increases motility in the abomasum and duodenum^64^. This finding suggests that the role of serotonin in enteric methane emissions from ruminants merits to be further investigated.

Several discriminant metabolites characteristically possess methylated groups such as betaine, stachydrine (also called proline-betaine), methylhistidine and the choline moiety of phosphatidylcholines. In addition, stachydrine is a derivative of pyrrolidine, another discriminant metabolite. Although we cannot draw further associations between these metabolites and enteric methane production, we suggest that they should be further scrutinized in future studies. Betaine and choline are readily converted by gut microbes into methylamines, particularly trimethylamine that is a known substrate of methylotrophic methanogens^65^. We and others had previously shown a negative relationship between methylated compounds in the rumen and urine on one hand and the methylotrophic Methanomassiliicoccales and methane emissions on the other hand^56,66^.

This study demonstrates the proof of principle that plasma metabolome reflects changes in enteric methane emissions in ruminants. In this work, 48 discriminant metabolites and 7 metabolic pathways were associated with the anti-methanogenic treatment. However, the development of proxies to predict methane emissions should involve a lower number of metabolites in order to be applicable under field conditions. A particular attention should be given to metabolites specifically provided by the microbiota or that can be linked to methanogenesis in order to develop enteric methane proxies based on plasma analysis. The number of animals used in this study does not allow choosing robust proxies but we explored the validity and predictive value of discriminant metabolites using logistic regression analyses. We first reduced the number of metabolites to 17. This was done by removing highly correlated metabolites and those metabolites involved in the same metabolic pathway (see “Methods”, Supplementary Table S6 and Supplementary Fig S1). Then, logistic regression was applied on various combinations of metabolites allowing to generate nine classification models with a similar predictive potential (See supplementary Table S7). According to the models, some metabolites seem to be major determinant of the phenotype (low/high methane producer). As stated above the number of animals used in this study implies that the predictive power of these models is limited but these results highlight the potential of the approach. A validation of both the approach and discriminant metabolites is required using a higher number of animals and different production conditions. Validation studies should be carried out on large populations to test the ability to detect natural variation within individual animals; also in studies using anti-methanogenic additives with different modes of action. The potential development of a methane emission proxy based on the analysis of selected plasma metabolites will need to take into consideration these validation steps.

## Conclusion

This study shows that variation in enteric methane emissions induced detectable changes in plasma metabolome in cattle. Some of the highlighted discriminant metabolites are from microbial origin and are known to be involved in methanogenesis or the utilization of hydrogen and electron acceptors in the rumen. Other discriminant metabolites found in plasma are produced by the host or have a mixed microbial-host origin. These latter metabolites are mostly involved in energy metabolic pathways and amino acids metabolism suggesting that a reduction in methanogenesis produced a general effect on the host animal. Plasma metabolites are useful to improve our understanding of the physiological effects on the host ruminant induced by a reduction of methanogenesis. Plasma could also be of interest for developing indirect alternative indicators of methane emissions from ruminants.

## Methods

The study was conducted at the animal facilities of the French National Institute of Agronomic Research (INRAE) UE1414 Herbipôle Unit (Saint-Genès Champanelle, France). Procedures were evaluated and approved by the French Ministry of Education and Research (APAFIS #2015073116475330) and carried out in accordance with French and European guidelines and regulations for animal experimentation.

### Animals and experimental procedures

Twenty-five Holstein primiparous dairy cows were used in this study. Animals were divided into two groups of equivalent days in lactation, dry matter intake (DMI), and milk yield (MY). Cows were fed with a total mixed ration of corn silage (30%), grass hay (30%), and concentrate (40%) containing (Treated; n = 12) or not (Control; n = 13) an anti-methanogenic additive (0.1% of the dry matter) (Supplementary Table S8). Cows were fed at 9 am and 4 pm and milked at 7.30 am and 3.30 pm. The experimentation lasted 6 weeks: 4 weeks of adaptation followed by 2 weeks for sampling and measurements. To allow for methane measurements (see below), cows were subdivided into four subgroups comprising similar number of Treated and Control cows that started the experimentation at 1 week intervals.

In week 5, blood samples were collected through the jugular vein using heparinized vacutainers (Greiner bio-one, Kremsmünster, Austria) (10 ml), in the morning after milking and before feeding. Blood samples were centrifuged (3,000 *g*; 15 min; 4 °C), and plasma was transferred into 1.5 ml polypropylene tubes and stored at − 80 °C until analysis. In week 6, 16 randomly chosen cows (eight per group) were transferred from free stalls into respiration chambers for measuring methane emissions for 4 days as previously described^67^. Briefly, groups of four cows (two Control and two Treated) were individually placed in open chambers for four consecutive days. The chambers operated at a slight negative pressure, with an air flow oscillating between 700 and 800 m^3^/h (about 45 air changes per h). Airflow in the exhaust duct of each chamber was continuously measured and automatically recorded every 5 min with a datalogger. Concentration of gases in the four chambers and in the outside ambient (background) was alternatively analyzed at a 0.1 Hz sample frequency for 5 min every 25 min using an infrared detector (Ultramat 6, Siemens, Karlsruhe, Germany) and recorded (Nanodac Invensys, Eurotherm Automation SAS, Dardilly, France). The detector was manually calibrated the day before each measurement period using pure N2 and a mixture of CH4 (650 ppm) and CO_2_ (700 ppm) in N2. Real-time gas emissions in a chamber were calculated by the difference between chamber and ambient gas concentrations multiplied by the airflow corrected for temperature, relative humidity and pressure according to the Wexler equation.

### Plasma metabolomics analyses

Plasma samples were analyzed by two untargeted complementary techniques (NMR and LC-QToF/MS) and one targeted LC–MS/MS to enhance the coverage of the metabolome^68,69^.

Nuclear Magnetic Resonance analysis was performed three weeks after plasma collection using the method described by Rohart, et al.^70^. Briefly, plasma samples (500 µl) were thawed on ice, 200 µl were transferred into a 1.5 ml polypropylene tube to which 500 µl of deuterium oxyde (D_2_O) phosphate buffer (pH 7) solution containing sodium trimethylsilyl propioniate (TSP, 1 mM) was added. Samples were vortexed, centrifuged (5,500 *g*; 10 min; 4 °C) and 600 µl of supernatant were transferred into NMR tubes for NMR spectroscopy analysis. The NMR analysis was performed on a Bruker Avance III HD spectrometer (Bruker, GMBH, Karlsruhe, Germany) operating at 600.13 MHz, and equipped with an inverse detection 5 mm ^1^H–^13^C–^15^N–^31^P cryoprobe connected to a cryoplatform. One dimensional (^1^H) spectra was acquired using a Carr-Purcell-Meiboom-Gill (CPMG) spin echo pulse sequence with a 5-s relaxation delay. A water signal suppression was achieved by presaturation during the relaxation delay. The spectral width was set to 20 ppm for each spectrum and 256 scans (16 dummy scans) were collected with 32 k points. Free induction decays were multiplied by an exponential window function (LB = 0.3 Hz) before Fourier Transformation.

For LC-QToF/MS analysis, plasma samples were analyzed five months after plasma collection following a procedure previously described by Pereira, et al.^71^ with slight modifications. Briefly, plasma samples (500 µl) were thawed on ice and 100 µl were transferred into 1.5 ml-tubes and mixed with 200 µl of cold methanol. After centrifugation (14,000 *g*; 10 min; 4 °C), supernatants were evaporated using a Genevac EZ-2 evaporator (Genevac SP Scientific, Ipswich, UK). Dried residues were solubilized in 50/50 (v/v) water/acetonitrile plus 0.1% formic acid mixture and analyzed in a ultra-performance liquid chromatography coupled to a quadrupole–time-of-flight mass spectrometer (Bruker impact HD2) equipped with an ESI source. In order to ensure performance of analytical instrumentation, quality control samples (QC) were prepared by pooling all extracted plasma samples and analyzed at the beginning of the sequence and then every five samples throughout the run.

The targeted method was based on electrospray ionization mass spectrometry measurements using the Absolute*IDQ* p180 kit assay (Biocrates Life Sciences AG, Innsbruck, Austria). The assay measures 188 metabolites belonging to seven different biochemical families: amino acids, biogenic amines, acylacarnitines, lyso-phosphatidylcholines, phosphatidylcholines, sphingolipids and monosaccharides (see supplementary Table S11).

### Data processing and statistical analysis

For NMR, spectra were manually phased and the baseline was corrected using TopSpin 3.2 software (Bruker, GMBH, Karlsruhe, Germany). All spectra were referenced to TSP signals at 0 ppm. The spectral data were imported in the Amix Software (version 3.9, Bruker, Rheinstetten, Germany) to perform data reduction in the region between 10.0 and 0.5 ppm with a bucket width of 0.01 ppm (n = 981 buckets). The region between 5.1 and 4.5 ppm corresponding to water signal was excluded and data were normalized to the total intensity of the spectra. A matrix containing chemical shifts and intensities was generated.

For LC-QToF/MS, raw data were converted into NETCDF files and processed using a Galaxy web instance workflow for metabolomics (W4M)^72^. Ions were extracted using the centWave algorithm (resolution = 10 ppm; peak width = 5 to 20; s/n = 5) included in the W4M XCMS platform^73^. Retention time correction was performed by applying peak-group algorithms and the minimum fraction of samples was set to 0.3. Data were filtered on retention time (< 0.4 min and > 22 min), fold (< 2) and tstat (< 0) to remove spurious signals followed by signal drift correction. Data were normalized using the linear method algorithm^74^. As for NMR, a matrix containing retention times, masses and intensities was generated.

Both NMR and MS data were then investigated using a combination of univariate and multivariate techniques. The former are based on conventional one-variable-at-a-time analysis, and still constitute the reference approach to evaluate the individual statistical relevance of specific metabolites. The latter allow metabolic profiles to be considered as a whole in the model. They are therefore particularly relevant in the context of untargeted metabolomics because of their ability to reveal potential relationships between subsets of compounds carrying common metabolic information, which could be overlooked otherwise. Before analysis, NMR data were log-Pareto scaled and LC-QToF-MS and targeted data were Unit Variance (UV) scaled (see supplementary Tables S9 to S10). The data were first investigated with multivariate techniques using SIMCA P software (V13, Umetrics AB, Umea, Sweden). Unsupervised Principal Component Analysis (PCA) was used to visualize trends and potential outliers (Supplementary Fig S2 and S3). Then, Supervised Orthogonal Partial Least Square Discriminant Analyses (OPLS-DA) was performed to find metabolites changes of variables as markers for methane production using the variable importance for projection (VIP) values. A variable selection step using multivariate analysis was applied for MS data and Orthogonal Signal Correction Partial Least Square Discriminant Analyses (OSC-PLSDA) for NMR data to reveal differences between Control and Treated groups. To validate these models, a permutation test based on 200 iterations was applied. Discriminant metabolites were subsequently subjected to univariate analysis (paired parametric test, FDR for multiple testing; p < 0.05) to identify the most significant among those selected.

### Metabolite identification

For NMR, identification was based on similarity of chemical shifts and coupling constants between plasma samples and standards. The comparison was performed between one dimensional analytical data and standards acquired under the same analytical conditions in our internal database, as well as from public databases like the Livestock Metabolome Database^75^ (LMDB, //www. https://lmdb.ca/) and the Biological Magnetic Resonance Data Bank^76^ (https://www.bmrb.wisc.edu/metabolomics/metabolomicsstandards.shtml). Identified chemical structures were confirmed using two-dimensional NMR experiments (Jres, COSY, HSQC) on pooled samples^77^.

For LC-QToF/MS data, discriminant metabolites were first selected (Level 1) using an in-house database containing more than 1,000 metabolites acquired under the same chromatographic and mass spectrometry conditions. Some of them were confirmed by comparing their retention time, exact masses and fragmentation spectra with those from standards acquired under the same chromatographic and fragmentation conditions. For the remaining unidentified metabolites, putative annotation was achieved by comparing their exact masses and/or their fragmentation spectra with those provided in public databases such as METLIN and HMDB. Fragmentation spectra were obtained on an Orbitrap Velos (Thermo, Les Ulis, France) at normalized collision energies (NCE) 25 in the ion trap (collision-induced dissociation (CID), resonant activation) as well as NCE 35 in a quadrupole collision cell (Higher-energy collisional dissociation (HCD), non-resonant activation). If needed, the collision energy was adapted for ion species with insufficient or too (important) harsh fragmentation (signal intensity of the precursor ion in the fragment spectra outside a 10–40% range). Chromatography were executed on UltraMate 3,000 (Dionex, Les Ulis, France), coupled to the Orbitrap Velos, using the same LC conditions as for the LC–MS acquisitions described before. Full scans were acquired at mass resolution 100,000 (FWHM, m/z 400) to obtain the exact mass whereas mass resolution 30,000 (FWHM, m/z 400) was used for fragmentation experiments, both with an AGC set at 1E6.

### Pathway analyses

The pathway analyses of discriminant metabolites (n = 48) was performed using MetaboAnalyst 4.0 (https://www.metaboanalyst.ca) and MetExplore 2.20 (https://metexplore.toulouse.inra.fr/metexplore2.20.12/). Up to 41 metabolites have Kegg identifiers that were mapped in MetaboAnalyst, whereas only 30 metabolites could be mapped using MetExplore. Identified discriminant metabolites were first analysed using MetPA in MetaboAnalyst 4.0 (https://www.metaboanalyst.ca). This tool allows to contextualize modification related to a phenomenon, in our case the reduction of methanogenesis based on pathway enrichment analysis and pathway topology analysis. The *Bos taurus* pathway was used and over representation analysis ^78^ was performed using a Fisher exact test. Topological analysis was based on the relative betweenness and out of degree centrality measure of a metabolite in a network. A combination of P value of 0.05 (1.2 = − log 0.05) on the pathway enrichment analysis and the pathway impact (= 0.2) was set to identify impacted metabolic pathways (Fig. 4).

Additionally, MetExplore^33^ was used to perform metabolomics data mapping and MetExploreViz^79^ for network visualization and sub-network extraction. The analysis was performed on Bos taurus KEGG (MetExplore Biosource id 2952) using KEGG identifiers (see supplementary Table S3). This network is built by gathering all reactions in KEGG for which at least one enzymatic gene is found in the annotated *Bos taurus* genome. Hence, some metabolites belonging to KEGG database may not belong to the *Bos taurus* network in MetExplore since there is no reaction with corresponding genes able to produce or consume them.

A sub-network extraction was applied using MetExplore algorithm (Fig. 5). The aim of the method is to extract from the entire metabolic network of *Bos taurus* (1826 reactions and 1526 metabolites) the reactions connecting metabolites of interest and analyze the network context. The output of the computation is useful to interpret changes in pathways and in the metabolome associated with the reduction of methane emissions. To do so the algorithm used consist in looking for the lightest path^80,81^ between each pair of metabolites. A path is a sequence of reactions connecting two metabolites, the path is weighted by the sum of the square of the degree of each metabolites in the path (the degree corresponds to the number of reactions producing or consuming a metabolite). The aim of the lightest path algorithm is to automatically retrieve the path with the lowest weight hence avoiding as much as possible highly connected metabolites like water or CO_2_. Subnetwork visualization was performed on Cytoscape^82^.

MetaboAnalyst and MetExplore are complementary as the former give information about pathways and the latter on the interconnection between metabolites within *Bos taurus* metabolic network.

### Predictive potential evaluation

To use the discriminant metabolites (n = 48) as proxies of methane emissions, their number should be refined. Logistic regression were used for exploratory purpose after removing redundant information. In that case, selection of metabolites was based on expert knowledge and statistical analysis (R^2^ > 0.7 and p < 0.05). Model selections were performed by choosing the best model amongst all the models that can handle up to 9 variables using the Akaike information criterion (AIC), and then completed with a forward and backward selection. Pearson correlation and Logistic regression were performed using Xlstat (XLSTAT 2017: Data Analysis and Statistical Solution for Microsoft Excel. Addinsoft, Paris, France). The visualization of the correlation was performed using Cytoscape.

## Supplementary information

Supplementary figures.

Supplementary tables.
