# Supplementary material for: Inhibition of enteric methanogenesis in dairy cows induces changes in plasma metabolome highlighting metabolic shifts and potential markers of emission
Source: Sci Rep. 2020 Sep 24;10:15591. doi: 10.1038/s41598-020-72145-w (PMC7515923; doi:10.1038/s41598-020-72145-w)
Supplement: Supplementary file 1 — Supplementary figures. [file 41598_2020_72145_MOESM1_ESM.docx]

**Inhibition of enteric methanogenesis in dairy cows induces changes in plasma metabolome highlighting metabolic shifts and potential markers of emission**

Bénédict Yanibada^1^, Ulli Hohenester^1^, Mélanie Pétéra^2^, Cécile Canlet^3,4^, Stéphanie Durand^2^, Fabien Jourdan^3^, Julien Boccard^5^, Cécile Martin^1^, Maguy Eugène^1^, Diego Morgavi^1*^, Hamid Boudra^1*^

**^1^**Université Clermont Auvergne, INRA, VetAgro Sup, UMR Herbivores, Saint-Genès-Champanelle, France,

**^2^**Université Clermont Auvergne, INRA, UNH, Plateforme d’Exploration du Métabolisme, MetaboHUB Clermont, F-63000 Clermont-Ferrand, France

**^3^**Toxalim, Research Centre in Food Toxicology, Université de Toulouse, INRA, ENVT, INP-Purpan, UPS, F-31027, Toulouse, France

**^4^**Axiom Platform, MetaToul-MetaboHUB, National Infrastructure for Metabolomics and Fluxomics, F-31027, Toulouse, France

**^5^**Institute of Pharmaceutical Sciences of Western Switzerland, University of Geneva, Geneva, Switzerland

**Correspondence**:

[abdelhamid.boudra@inra.fr](mailto:abdelhamid.boudra@inra.fr)

Phone: + 33 4 73 62 41 06

[diego.morgavi@inra.fr](mailto:diego.morgavi@inra.fr)

Phone: + 33 4 73 62 40 57

Supplementary Fig S1. High correlation between discriminant metabolites


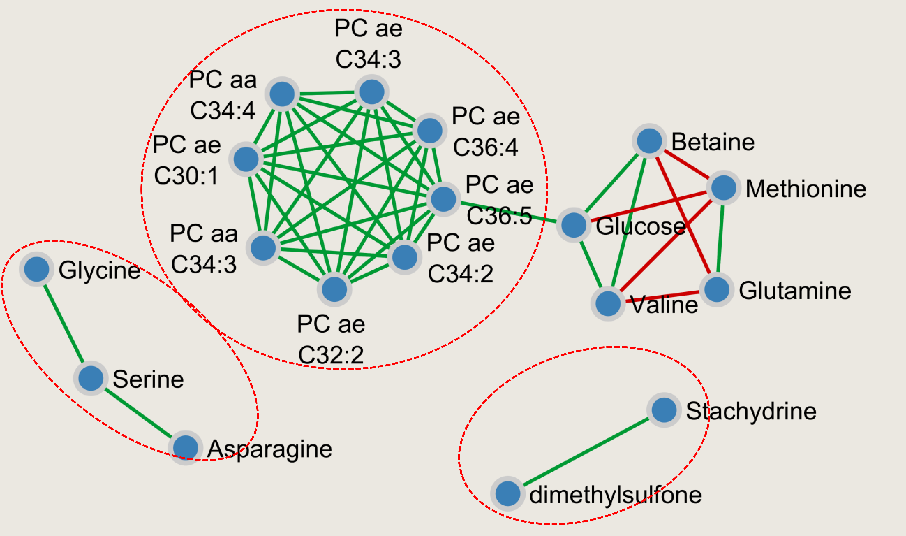


Graphical representation of highly correlated metabolites (threshold=0.7 with a maximum pvalue=0.001) obtained in MetaboAnalyst 4.0 (<http://www.metaboanalyst.ca>). Each nodes (blue) represents a metabolite (n=18) and each edge a correlation (green positively correlated; red= negatively correlated). Highly correlated metabolites likely convey similar information and have similar weight on any logistic regression model. It is not usefull to kept redundant information.

Supplementary Fig S2. ACP of the NMR data


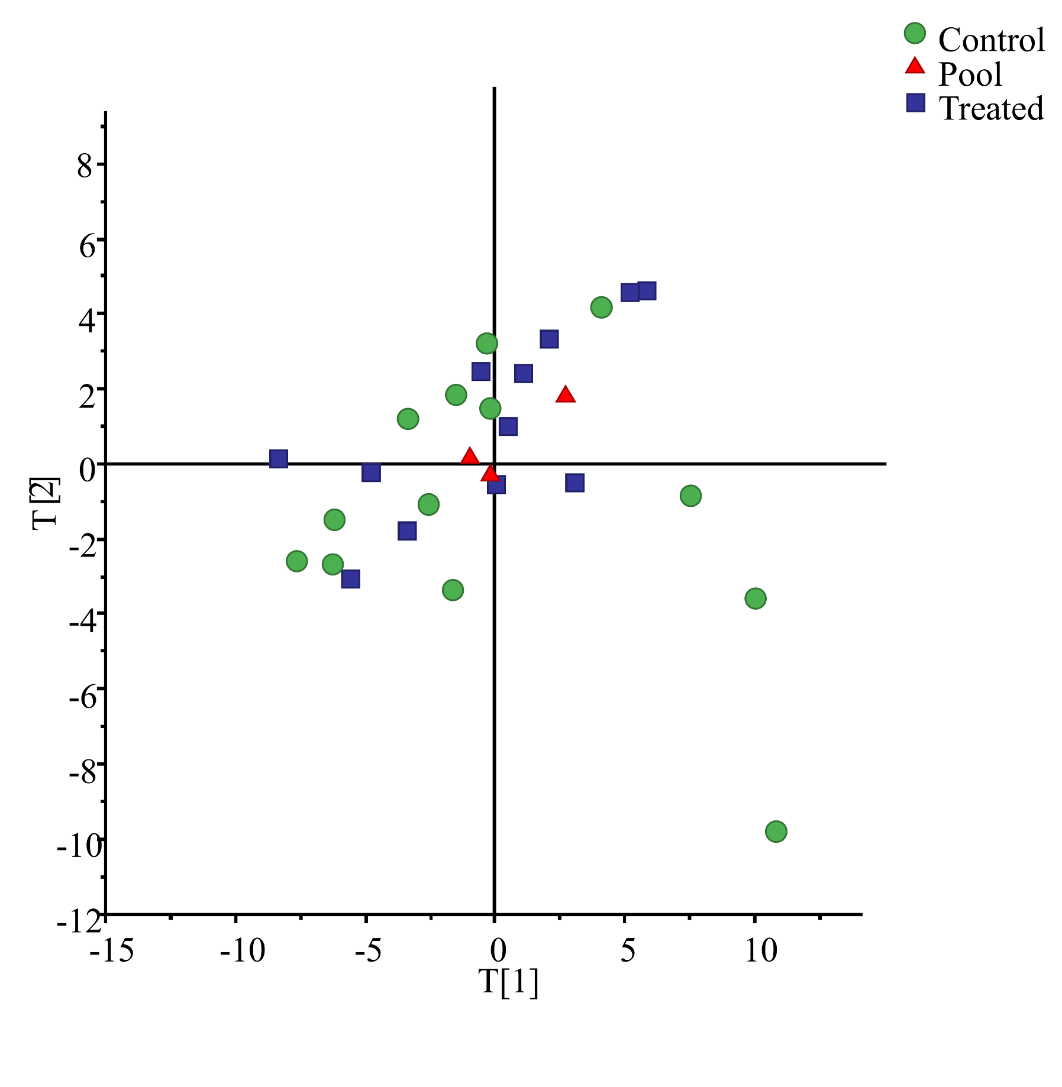


Principal component analysis of the NMR data. (n=4; R^2^X1=0.286; R^2^X2=0.110; R^2^X=0.547). (Green circle=Control group; Blue square=Treated group; red triangle=pool).

Supplementary Fig S3. ACP of the LC-QToF-MS data


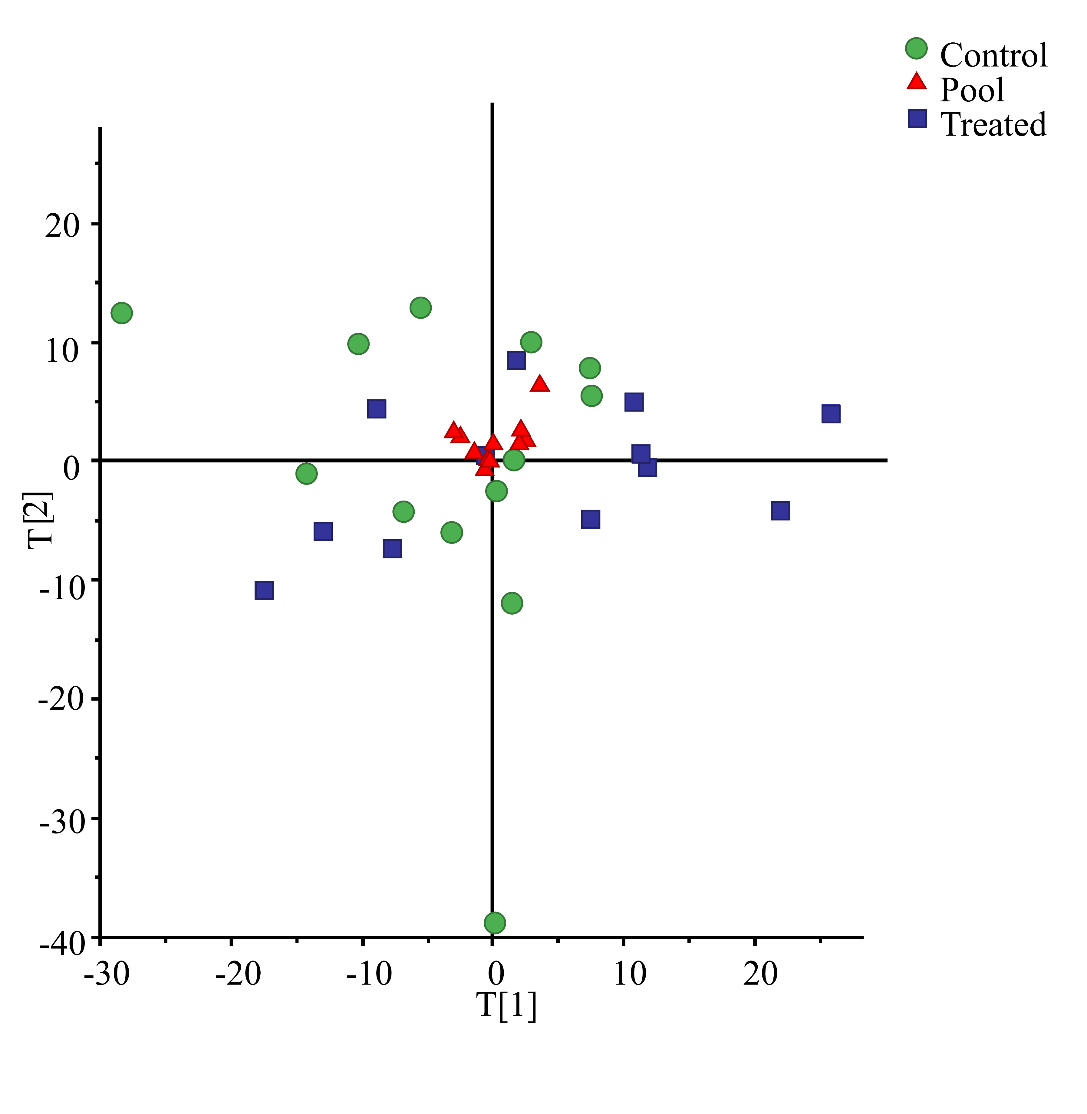


Principal component analysis of the LC-QToF-MS data. (n=7; R^2^X1=0.132; R^2^X2=0.103; R^2^X=0.512). (Green circle=Control group; Blue square=Treated group; red triangle=pool).
